# Cumulative incidence, distribution, and determinants of catastrophic health expenditure in Nepal: results from the living standards survey 

**DOI:** 10.1186/s12939-018-0736-x

**Published:** 2018-02-14

**Authors:** Mamata Ghimire, Rakesh Ayer, Masahide Kondo

**Affiliations:** 1 Department of Health Care Policy and Management, Graduate School of Comprehensive Human Sciences, University of Tsukuba, Tennoudai, Tsukuba, Ibaraki, Japan; 2 Department of Community and Global Health, Graduate School of Medicine, The University of Tokyo, Tokyo, Japan; 3 Department of Health Care Policy and Health Economics, Faculty of Medicine, University of Tsukuba, Tennoudai, Tsukuba, Ibaraki, Japan

**Keywords:** Catastrophic health expenditure, Incidence, Distribution, Determinants, Nepal

## Abstract

**Background:**

Nepal has committed to the global community to achieve universal health coverage by 2030. Nevertheless, Nepal still has a high proportion of out-of-pocket health payment and a limited risk-pooling mechanism. Out-of-pocket payment for the healthcare services could result in catastrophic health expenditure (CHE). Evidence is required to effectively channel the efforts to lower those expenses in order to achieve universal health coverage. However, little is known about CHE and its determinants in a broad national context in Nepal. Therefore, this study was conducted to explore the cumulative incidence, distribution, and determinants of CHE in Nepal.

**Methods:**

Data were obtained from the nationally representative survey, the Nepal Living Standards Survey-third undertaken in 2010/11. Information from 5988 households was used for the analyses. Households were classified as having CHE when their out-of-pocket health payment was greater than or equal to 40% of their capacity to pay. Remaining households were classified as not having CHE. Logistic regression analyses were used to identify determinants of CHE.

**Results:**

Based on household-weighted sample, the cumulative incidence of CHE was 10.3% per month in Nepal. This incidence was concentrated in the far-western region and households in the poorer expenditure quartiles. Multivariable logistic regression revealed that households were more likely to face CHE if they; consisted of chronically ill member(s), have a higher burden of acute illness and injuries, have elderly (≥60 years) member(s), belonged to the poor expenditure quartile, and were located in the far-western region. In contrast, households were less likely to incur CHE when their household head was educated. Having children (≤5 years) in households did not significantly affect catastrophic health expenditure.

**Conclusions:**

This study identified a high cumulative incidence of CHE. CHE was disproportionately concentrated in the poor households and households located in the far-western region. Policy-makers should focus on prioritizing households vulnerable to CHE. Interventions to reduce economic burden of out-of-pocket healthcare payment are imperative to lower incidences of CHE among those households. Improving literacy rate might also be useful in order to lower CHE and facilitate universal health coverage.

**Electronic supplementary material:**

The online version of this article (10.1186/s12939-018-0736-x) contains supplementary material, which is available to authorized users.

## Background

Paying for the healthcare services out-of-pocket (OOP) poses adverse impacts on household economy. Millions of households struggle to finance their healthcare expenses and many of them are driven below the poverty line by such expenses [1, 2]. This struggle for healthcare expenditure is more pronounced in low-income countries where the health system is poorly funded. In those health systems, measures to protect households financially from healthcare expenditure, such as risk-pooling mechanisms,^1^ are either absent or inadequate [3]. Therefore, in the absence of a financial safety net, healthcare should be purchased by OOP [3]. Any OOP healthcare expenditure that exceeds a specified threshold of household spending is catastrophic and is also referred as catastrophic health expenditure (CHE) [4, 5]. Financial risk protection from CHE is a key target of universal health coverage (UHC), as mandated by the sustainable development goals (SDGs) [6]. Evidence shows, the financial capacity of households to maintain their basic needs is jeopardized due to CHE [7]. Households incurring CHE are likely to compromise their children’s education [8], sell assets [9], and pushed into poverty [10].

The greater episodes of CHE are associated with the higher proportion of OOP payments in the total health expenditure at the health system level [4]. Reversely, OOP payment in health is found to decline when government expenditure in healthcare increases [1]. The share of the government health expenditure is lowest in low-income countries particularly from the South Asia [1]. Despite a call of UHC to protect households from CHE, OOP payment still makes almost half of total health expenditure in those countries [11].

Nepal is a low-income country in South Asia. In Nepal, limited risk-pooling in healthcare makes financial protection no better than most other low-income countries. The health financing system in Nepal does not ensure to protect households from CHE [12]. Despite the commitment of the Nepalese Government (GoN) to achieve UHC by 2030, nearly 48% of total health expenditure is still financed by OOP payments [13]. Some of the major public subsidies present in Nepal are treatment subsidies for chronic illness, essential free health care scheme, and cash incentives to pregnant women [14]. However, due to fragmented nature of those subsidies and absence of comprehensive health insurance, necessary financial protection in healthcare is not achieved [12]. Voluntary community-based health insurance programs are present but have low-enrollment rate [15]. Absence of comprehensive health insurance mechanism, high morbidity, reliance on OOP in financing treatment and large share of poverty [16] might have exposed Nepalese households to an increased risk of CHE.

In the past, a few attempts were undertaken to measure the household healthcare expenditure in Nepal [17–20]. Using 1994/95 Nepal Living Standards Survey data, one of the earlier studies examined OOP healthcare payment in Nepal [18]. The study found that financing healthcare by OOP was the major economic burden to the Nepalese households. However, that study did not extend its analyses to CHE. Similarly, a multi-country study also used the same data to report the incidence of CHE in Nepal [17]. That study did not explain the CHE variation by rural/urban or regional context. Other existing studies on CHE in Nepal are either disease-specific [19] or place-specific, that is, conducted in Kathmandu Valley- urban location [20]. Those previous studies may have provided incomplete national scenario on CHE.

We are still unaware of distribution and determinants of CHE in a larger social and economic context. For example, the variation in CHE by administrative regions or by household’s economic condition and demographic composition. These contexts are shown to be important by the international studies [7, 21–23]. This study aims to contribute to the existing literature by providing answers to these questions by using the latest available nationwide survey data. Policy-wise, knowledge of distribution and determinants is essential to protect the vulnerable households from CHE. It is equally helpful to design the efficient healthcare financing system of the country. Therefore, with the intention to fill the data gap, this study had three objectives: first, to investigate the national cumulative incidence of CHE; second, to measure the distribution of CHE; and final, to examine the determinants of CHE in Nepal.

## Methods

### Data source

This study utilized cross-sectional survey data from the nationally representative Nepal Living Standards Survey-third (NLSS-III). NLSS-III was undertaken by the Central Bureau of Statistics- Nepal (CBS) in 2010/11. This is the latest publicly available Living Standards Survey in Nepal. NLSS-III divided 75 (total) districts of Nepal into 14-strata and each stratum was assigned to primary sampling units (PSUs). The PSUs were selected with probability proportional to size, where a number of the household was the measure of size. PSUs were either a ward or a sub-ward in the village development committee. The household was a survey unit.

Data collection for NLSS-III was done over a period of 12 months which was divided into 4 phases to capture the seasonal variation. The survey covered all the administrative regions and geographical belts of the country. Data were collected by a) face-to-face interview, b) observation notes by trained research assistants, and c) anthropometric measurements. Data accuracy of survey was maintained by spot-checks and re-checks. A total of 5988 household data collected from 499 PSUs was included in the dataset for the public use. We obtained NLSS-III data from the CBS. All variables used in this study had complete data except for housing rent. Housing rent together with other household expenses was used to compute the total household expenditure. Very small number of household (269 out of 5988 (0.04%)) had missing data on housing rent. Such missing data was imputed by using a hedonic regression model. The method of this survey is detailed elsewhere [24].

### Contents of survey questionnaire

The NLSS-III household questionnaire contained 21 sections and 9 appendices. We chose five sections among those for this study. First, household roster section was used to gather household demography information. Second, housing section for information on a household’s housing expenses. Third, access to facilities section to compute accessibility to the nearest health facility. Fourth, food and non-food expenditure section for the estimation of household consumption expenditure. Final, the health section to analyse treatment costs of illness and injuries. Monetary and in-kind payments made by households on food and non-food items were aggregated to obtain household consumption expenditure as recommended [25]. Recall period was 7 days, 30 days, and 12 months for food expenditure, non-food expenditure, and infrequent non-food expenditure, respectively. We adjusted all data into a 30-days figure as we aimed to measure monthly expenses of households.

In the health section, respondents were asked to report the latest episode of acute illness and injury they suffered during 30 days before the interview. Their responses were coded into 14 categories. Chronic illness was defined as an illness lasting for more than a year. The primary chronic illness suffered and reported by respondents was coded into 13 groups. Any cost, monetary and in-kind, spent for the treatment of chronic illness (in last 12 months) and acute illness and injuries (in last 30 days) was recorded. Incurred expenses by households were recorded in Nepali Rupees (NRs).

### Measurement of CHE

Two major approaches to measure CHE are available [26, 27]. Firstly, OOP is measured as the proportion of household’s total income (say ‘Y’); if OOP/Y exceeds the pre-determined threshold, for example 2.5% to 15%, the healthcare expenditure is catastrophic [26]. This method is criticised for the equity reason as it uses the same threshold value for both the poor and rich household. The rich households are more likely to overshoot the threshold level without suffering much adverse effect mostly at the higher threshold [27]. Secondly, OOP is measured as the proportion of household’s capacity to pay (CTP) put forward by Xu et al. [4]. CTP = Y-*se*; where ‘Y’ is the income (or consumption expenditure) used in the first approach. The *se* is the basic requirement to maintain a life. A poverty line is used to analyse *se.* If OOP/CTP is equal to or more than 40% of CTP healthcare expenditure is catastrophic. We took 40% cut-off point because this value is recommended by Xu et al. [4] and is used in previous studies [21–23]. This approach is said to have addressed the equity issue created by the first approach [27]. We used the second approach.

In the second approach, series of steps are involved in the measurement of CHE. These steps were recommended in a multi-country study [4] and extensively used by previous studies [21–23]. The multi-country study was based on the household survey data of 59 countries. Countries from the South Asia region were also involved in that multi-country study. In the same study, the value of β (a household scale multiplier) used was 0.56 (95% CI 0.556–0.572), and was obtained in from a regression equation based on those 59 countries: ln(food expenditure) = ln(k) + β ln(household size) + ∑ γicountry. We used β = 0.56 as the part of the recommendation of the multi-country study.

The steps involved in CHE measurement (see Appendix for detailed explanation) are;

Out-of-pocket *(OOP)* health payment: direct payment made by households at the point of service use. Payment comprised fee for registration, diagnosis, consultation, surgery; medicine and transportation cost.

Household consumption expenditure *(hh_exp)*: included both monetary and in-kind payment on all goods and services and the money value of the consumption of home-made products.

Food expenditure *(foodh)*: included food expenses and the value of own production by the household.

Equivalent scale *(eqsize):* calculated as*, eqsize* = *hhsize*^*β*^, was used (hhsize = household size).

Equivalent food expenditure *(eqfoodh)* calculated as, *eqfoodh* = *foodh*/*eqsize*

Poverty line *(pl):* calculated from among the survey households whose food expenditure was an average of food expenditure of households in 45^th^ and 55^th^ percentile of the total sample. *pl* = average of eqfoodh where foodh 45 < *eqfoodh* < *foodh* 55

Subsistence spending *(se)*: the minimum requirement to maintain basic life, se = pl ∗ *eqsize*

Capacity to pay *(CTP)*: non-subsistence expenditure by households. For households reporting food expenditure lower than *se*, CTP was defined as total expenditure minus food expenditure. *CTP* = *hh*_ *exp*  – *se  if se* <  = *foodh*; *CTP* = *hh*_ *exp*  – *foodh  if se* > *foodh*

Catastrophic health expenditure *(CHE):* calculated as, CHE = 1 if OOP/CTP >  = 0.4, otherwise CHE = 0

### Variables selection

#### Independent variable

Published studies from low- and middle- income countries offered some guidance in the selection of independent variable in our search for determinants of CHE for this study. Poorer households [22, 28], household demography (≤ 5 year children, ≥ 60 years elderly) [29, 30], urban location [23], female household head [21], chronic illness [31, 32] and increased illness episodes [33] were positively associated with CHE. However, households with educated household head were less likely to have CHE [31]. We also extracted variables such as geographical belts and administrative regions with the intention to explore the distribution of CHE. From north-to-south transect, Nepal has three geographical belts, mountain, hill, and tarai. These belts represent ecological variation. Administratively, Nepal is divided into five development regions- eastern, central, western, mid-western, and far-western from the east to the west. These administrative regions are not proportionately developed. For example, the central region is prosperous than other regions [16]. From a policy perspective, evidence of the regional distribution of CHE could be of importance to Nepal. Particularly, we were interested to see the variation of CHE by household demography, illness and injury burden, regional and geographical location, settlement area, and economic condition.

Existing studies have shown that catastrophic health expenditure depends on household’s socio-demographic and economic factors, particularly, household demographic composition, literacy and economic status [22, 29–32]. However, it is important to know if those socio-demographic and economic variables interact with each other to produce a biased result. In this study, we introduced three sets of interaction terms to see how those socio-demographic and economic factors mediate with each other and with dependent variable, CHE. ‘Household with at least one under 5-years child x Household reporting acute illness and injuries’ was the first interaction term. Similarly, ‘Household with at least one 60-years and above elderly x Household reporting chronic illness’ was another pair of interaction term. Finally, ‘Expenditure quartiles x Literate household head’ was the last pair of interaction term included in the model.

#### Dependent variable

CHE was the dependent variable in this study.

#### Variables extraction and computation

Variables were extracted at the individual and household level. Raw survey data were used to extract information on individuals reporting chronic illness, acute illness and injuries, seeking care for their acute illness and injuries. A number of individuals reporting both illness was computed. At the household level; gender, education of household head, settlement area, and commute time to the nearest health facility was extracted from the raw data. OOP health expenditure, OOP share of household expenditure, and CTP were computed. Expenditure quartile was ranked by the equivalized per capita household consumption within the sample size. We also calculated household illness ratio for our analysis. The actual household size instead of equivalent size was used for the calculation on illness ratio.

### Statistical analyses

First, we summarized descriptive statistics of extracted and computed variables. Descriptive statistics results were used to see the distribution of the cumulative incidence of CHE among the expenditure quartiles and administrative regions. Second, we assessed the relationship between independent and dependent variables by univariate logistic regression. Finally, we performed multivariable logistic regression. With the exception for ratio of total illness episodes, all variables were taken for univariate logistic regression and then subsequently fed into multivariable logistic regression analysis (34). We saw acute illness and injury ratio and chronic illness ratio of a household separately as we were interested to see their influence on CHE. Household weight was used for the data analysis. All analyses were performed using STATA version 13.1 (College Station, Texas, USA).

## Results

Table 1 gives the summary statistics of extracted and computed variables from the NLSS-III. Around 10.2% of individuals reported chronic illness while 18.7% reported acute illness. The mean monthly household expenditure was approximately NRs. 30,000 (414.1 US$) and the OOP healthcare expenditure was NRs. 1187 (16.4 US$). The average household size was 4.9 ± 2.3. Less than half of households had ≤5 year children, and so were households with elderly members. Almost three-fourth of household heads were male and only one-fourth of the household heads were literate. Geographically, the proportion of households in mountain, hill and tarai belt consisted of 6.9%, 47.4% and 45.7%, respectively. Region-wise, the central region consisted the highest proportion of households (35.7%) while the far-western region consisted the least proportion of households (8.5%). Likewise, 73.1% of households commuted less than 60 min to reach the nearest health facility. Of the total households, 43.0% reported chronic illness, 59.4% reported acute illness, and 27.2% households reported both illnesses. The household burden of acute illness and injuries was greater than that of chronic illness. The proportion of households incurring CHE was 10.3% per month.

**Table 1:** Descriptive statistics of variables extracted and computed from the Nepal Living Standards Survey (NLSS)- Third, 2011

Variable description	Observation (N)	Weighted			Unweighted		
		Proportion	Mean	SD	Proportion	Mean	SD
Extracted Variables							
Individual Level							
Individuals reporting chronic illness [yes = 1; otherwise,0]	28,474	10.2%			11.4%		
Individuals reporting acute illness and injuries ^a^ [yes = 1; otherwise,0]	28,474	18.7%			19.4%		
Individual who sought care for their acute illness and injuries [yes = 1; otherwise,0]	5518	70.7%			69.3%		
Individual weights	28,670					5891.5	3717.9
Household Level							
Household expenditure ^b^ (NRs ^c^) (in 1000)	5988		30	82		33	99
Out of pocket health expenditure (NRs)	5988		1187	4657		1175	4743
Household size	5988		4.9	2.3		4.8	2.3
Household has under 5-years children [yes = 1; otherwise,0]	5988	41.7%			39.9%		
Household has elderly (60 years and above) population [yes = 1; otherwise,0]	5988	33.2%			31.8%		
Household head is male [yes = 1; otherwise,0]	5988	73.4%			73.3%		
Literate household head [yes = 1; otherwise,0]	5988	22.8%			25.9%		
Settlement area is urban [yes = 1; otherwise,0]	5988	20.9%			34.9%		
Geographical belts	5988						
Mountain		6.9%			6.8%		
Hill		47.4%			53.5%		
Tarai		45.7%			39.7%		
Administrative regions	5988						
Eastern		23.5%			21.3%		
Central		35.7%			38.1%		
Western		20.1%			19.2%		
Mid-western		12.2%			12.6%		
Far-western		8.5%			8.8%		
Commute time to the nearest health facility is less than 1 h [yes = 1; otherwise,0]	5988	73.1%			63.4%		
Households reporting chronic illness [yes = 1; otherwise,0]	5988	43.0%			41.7%		
Households reporting acute illness and injuries [yes = 1; otherwise,0]	5988	59.4%			56.9%		
Household weights	5988					964.6	348.3
Computed Variables							
Individual Level							
Individuals reporting both chronic and acute illness [yes = 1; otherwise,0]	28,670	30.5%			26.3%		
Household Level							
Capacity to Pay (NRs) (in 1000)	5988		18	82		22	99
OOP share of household expenditure (%)	5988		3.9	8.0		3.7	7.8
OOP share of household capacity to pay	5988		44.6	17.4		42.1	17.2
Equivalent household size for each household	5988		2.4	0.6		2.3	0.6
Households reporting both chronic and acute illness [yes = 1; otherwise,0]	5988	27.2%			25.6%		
Number of family members with chronic illness episodes	5988		0.6	0.7		0.5	0.7
Number of family members reporting acute illness and injuries episodes	5988		1.0	1.1		0.9	1.1
Ratio of acute illness and injuries episodes to household size	5988		0.2	0.2		0.2	0.2
Ratio of chronic illness episodes to household size	5988		0.1	0.2		0.1	0.2
Ratio of total illness episodes to household size	5988		0.4	0.4		0.4	0.4
Expenditure quartiles ^d^	5988						
1		25.0%			22.6%		
2		25.0%			22.8%		
3		25.0%			24.9%		
4		25.0%			29.7%		
Households with CHE (> = 40% of CTP) [yes = 1; otherwise,0] ^e^	5988	10.3%			9.1%		

^a^recall period was 30 days and only one episode was reported. ^b^ expenditures were adjusted in a monthly figure. ^c^ in February 2011, 1US$ was approximately equal to 72.45NRs. ^d^ quartile 1 represents the poorest while 4 represents the wealthiest. ^e^Cumulative incidence of catastrophic health expenditure (CHE) per month

**Fig. 1:**
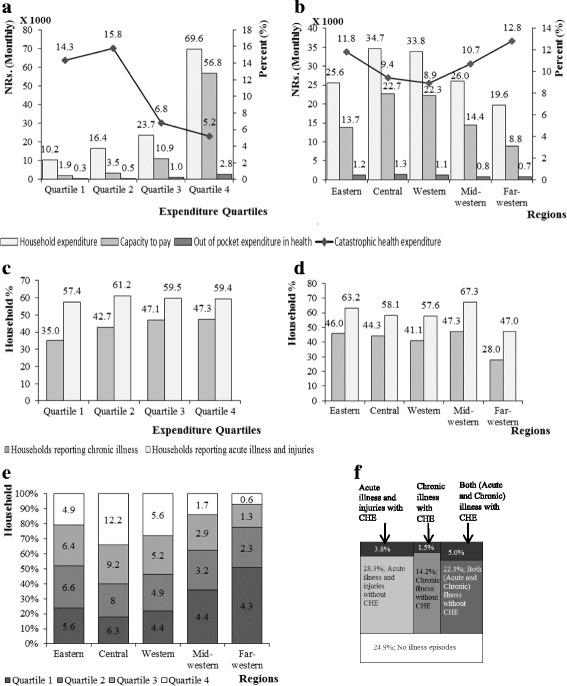
Distribution of financial characteristics, illness, and CHE across expenditure quartiles and administrative regions in Nepal, 2011. This figure consists of six sub-figures, (**a**-**f**), to reflect the nationwide distribution of CHE. Each sub-figure has unique title. **A.** Distribution of financial characteristics and CHE across expenditure quartiles. **b** Distribution of financial characteristics and CHE across administrative regions. **c** Distribution of illnesses across expenditure quartiles. **d** Distribution of illnesses across administrative regions. **e** Distribution of expenditure quartiles across administrative regions. **f** National picture of reported household illness episodes with and without CHE

Figure 1 is a multi-panel figure and consists of six figures, A to F. The Fig. 1 shows the distribution of financial characteristics, illness, and CHE across expenditure quartiles and administrative regions. Figure A shows that the increasing order of household expenditure, CTP, and OOP health expenditure as we move from the 1st quartile (poorest) to the 4th quartile (wealthiest). However, CHE was highest (15.8) in the 2nd quartile. Figure B demonstrates that the far-western region had the lowest CTP and OOP but the highest CHE (12.8). Figure C shows that the households in the 2nd quartile and 4th quartile reported the highest percentage of acute illness (61.2) and chronic illness (47.3) respectively. Figure D presents regional distribution of illness. Households in the eastern and mid-western regions reported a higher proportion of illness and injuries. Figure E further shows that the far-western and mid-western regions mostly consisted of households belonging to the poor quartiles in comparison to other regions. Figure F shows the proportion of reported household illness with and without CHE. Of the total, 75.1% of households reported illness and 10.3% of those illness episodes were with CHE. Similarly, for the households reporting chronic illness episodes only, 1.5% experienced CHE. This proportion was higher (3.8%) in case of households reporting acute illness and injuries. Likewise, 5.0% of episodes were with CHE in the households reporting the double burden of illness.

Table 2 shows univariate logistic regression of independent variable against dependent variable, CHE. Households having elderly member(s) and children were vulnerable to CHE. However, households in urban and households with educated heads were less likely to incur CHE. Households reporting chronic illness, acute illness and injuries, and a higher ratio of illnesses, in the hilly belt, in 1st and 2nd quartiles, were more likely to suffer from CHE. All variables from univariate logistic regression were fed into multivariable logistic regression.

**Table 2:** Univariate logistic regression of household characteristics with CHE (*N* = 5988)

Household Characteristics	CHE	
	OR (95% CI)	*p*-value
Equivalent household size	0.97(0.83–1.14)	0.736
Household has under 5-years children [yes = 1; otherwise,0]	1.22(1.00–1.49)	0.048
Household has elderly (60 years and above) population [yes = 1; otherwise,0]	1.60(1.34–1.92)	< 0.001
Household head is male [yes = 1; otherwise,0]	0.90(0.72–1.11)	0.334
Settlement area is urban [yes = 1; otherwise,0]	0.46(0.35–0.60)	< 0.001
Literate household head [yes = 1; otherwise,0]	0.51(0.39–0.68)	< 0.001
Commute time to the nearest health facility is less than 1 h [yes = 1; otherwise,0]	1.85(1.46–2.34)	< 0.001
Households reporting at least one member with chronic illness [yes = 1; otherwise,0]	2.48(2.04–3.03)	< 0.001
Households reporting at least one member with acute illness and injuries [yes = 1; otherwise,0]	4.68(3.64–6.01)	< 0.001
Chronic illness ratio	4.14 (2.89–5.77)	< 0.001
Acute illness and injuries ratio	6.49 (4.94–8.52)	< 0.001
Expenditure quartiles		
1	3.02(2.24–4.08)	< 0.001
2	3.40(2.59–4.47)	< 0.001
3	1.16(0.52–1.64)	0.374
4	1.00	
Geographical belts		
Mountain	1.00	
Hill	0.66(0.45–0.98)	0.040
Tarai	0.98(0.67–1.44)	0.925
Administrative regions		
Central	1.00	
Eastern	1.28(0.97–1.69)	0.075
Western	0.94(0.69–1.29)	0.718
Mid-western	1.14(0.83–1.57)	0.398
Far-western	1.40(0.99–2.00)	0.055

CHE is the dependent variable (CHE = 1 if OOP/CTP > =40%, otherwise CHE = 0)

Table 3 presents the determinants of CHE. Model 1 is the multivariable logistic regression without any interaction terms. Model 1 shows that households with elderly members (33.2%) were more likely to experience CHE. Whereas, households with a literate household head (22.8%) were less likely to incur CHE. Households with chronic illness, acute illness were all highly likely to face CHE. The acute illness burden of a household was catastrophic (OR = 2.33, 95% CI = 1.48, 3.67) than the chronic illness burden. Households with the poor economic condition incurred CHE. The findings revealed that households in the far-western region were vulnerable to CHE (OR = 1.46, CI = 1.02, 2.11) in comparison to households in the central region. Model 2 in Table 3 introduces three sets in interaction terms. None of the interaction terms were statistically significant. Selected interaction terms did not significantly influence either independent variables used in this study or their main effect on dependent variable as presented in model 1.

**Table 3:** Determinants of CHE in Nepal, 2011

Household Characteristics	Model 1	Model 2
	OR (95% CI)	OR (95% CI)
Equivalent household size	0.87(0.71–1.07)	0.88(0.72–1.08)
Household has under 5-years children [yes = 1; otherwise,0]	1.11(0.87–1.41)	0.87(0.54–1.41)
Household has elderly (60 years and above) population [yes = 1; otherwise,0]	1.37(1.13–1.66)**	1.34(0.96–1.87)*
Household head is male [yes = 1; otherwise,0]	1.00(0.79–1.27)	1.00(0.79–1.27)
Settlement area is Urban [yes = 1; otherwise,0]	0.79(0.58–1.06)	0.79(0.58–1.06)
Literate household head [yes = 1; otherwise,0]	0.73(0.54–1.00)*	0.61(0.34–1.10)*
Commute time the nearest health facility less than 1 h [yes = 1; otherwise,0]	1.05(0.79–1.38)	1.03(0.78–1.36)
Households having member with chronic illness [yes = 1; otherwise,0]	2.40(1.78–3.24)***	2.37(1.72–3.29)***
Households having member with acute illness [yes = 1; otherwise,0]	3.41(2.53–4.58)***	3.07(2.15–4.37)***
Acute illness ratio	2.33(1.48–3.67)***	2.39(1.52–3.76)***
Chronic illness ratio	1.27(0.65–2.48)	1.25(0.64–2.44)
Expenditure quartiles		
1	3.15(2.21–4.49)***	2.91(1.98–4.27)***
2	3.29(2.42–4.49)***	3.14(2.23–4.45)***
3	1.05(0.73–1.52)	1.07(0.71–1.59)
4	1.00	1.00
Geographical belts		
Mountain	1.00	1.00
Hill	0.92(0.62–1.38)	0.92(0.62–1.38)
Tarai	1.21(0.81–1.80)	1.21(0.81–1.80)
Administrative regions		
Central	1.00	1.00
Eastern	1.02(0.76–1.37)	1.03(0.77–1.37)
Western	0.93(0.68–1.27)	0.93(0.68–1.27)
Mid-western	0.86(0.63–1.16)	0.86(0.63–1.16)
Far-western	1.46(1.02–2.11)*	1.47(1.02–2.11)*
Interaction terms		
Household with at least one under 5-years child x Household reporting acute illness and injuries		1.32(0.78–2.21)
Household with at least one 60-years and above elderly x Household reporting chronic illness		1.02(0.67–1.57)
Expenditure quartiles x Literate household head		
Poorest (1) x Yes		1.89(0.02–4.38)
Quartile 2 x Yes		1.20(0.57–2.54)
Quartile 3 x Yes		0.83(0.34–2.06)
Wealthiest (4) x Yes		1.00

Model 1: without interaction terms. N = 5988, LR χ^2^ = 599.1, Pseudo R^2^ = 0.1524, Mean VIF = 2.01. Model 2: with interaction terms. *N* = 5988, LR χ^2^ = 564.6, Pseudo R^2^ = 0.1539, Mean VIF = 2.38

*p < 0.10

**p < 0.01

***p < 0.001

Multicollinearity was not identified in multivariable logistic regression model 1 and model 2. This conclusion was reached after calculating variance inflation factor (VIF). The mean VIF was 2.01 for model 1 and 2.38 for model 2. None of the variables had variance inflation factor greater than 10.0, the recommended value [34]. Besides that, goodness of fit test the model 1 was *p* = 0.102 and model 2 was *p* = 0.106. We checked this by Hosmer- Lemeshow goodness of fit test. The diagnostic plots of the model are provided in Additional file 1.

## Discussion

This study was based on nationally representative data and the findings are important as they provide a complete national scenario on CHE in Nepal. The study answers its three research questions. First, the cumulative incidence of CHE was 10.3% per month at the national level in Nepal. Second, CHE was distributed unevenly across expenditure quartiles and administrative regions. Third, CHE was determined by household illness, economic condition, and location. For instance, households with chronically ill member(s), higher episodes of acute illness, located in the poorer quartiles and the far-western region were more likely to face CHE.

The national cumulative incidence of CHE was 10.3% per month at a threshold of 40% or greater household’s CTP. Chronic illness was reported by 43.0% households. Similarly, the incidence of acute illness and injury was 59.4%. In the poorly developed risk-pooling system, seeking treatment to this staggering burden of illness might have imposed high healthcare cost to households. Evidence shows that risk-pooling mechanism such as insurance offers financial protection against CHE [35]. UHC targets to achieve 100% financial protection against CHE [6]. In this context, 10.3% cumulative incidence of CHE per month is high and needs an immediate attention.

CHE was distributed disproportionately in the poor quartiles and regions. Across expenditure quartile, CHE was concentrated in the 1st and 2nd quartiles in comparison to others. This concentration could be explained by household CTP. The 4th quartile had a higher average CTP, almost 28 times, as compared to the 1st. Due to low CTP, OOP payment for illness carried potential to be the catastrophic expenditure to those households. A similar trend was shown by previous studies [21, 23]. Region-wise, the highest incidence of CHE was seen in the far-western followed by the eastern. However, households in the far-western region were hit hardest by CHE as 77.6% of households in the far-western belonged to the poorer quartiles compared to 51.9% in the eastern region.

Illness burden in household determined CHE in Nepal. Households with chronically ill member(s) were 2.4 times more likely to suffer CHE. Chronic illness often needs continuous treatments and consultations. This puts households in constant pressure to finance such treatment which invites financial ruins. The result supports findings from previous studies [28, 33]. This reason also offers the explanation on the increased risk of CHE among households with elderly member(s). Households reporting at least one member with acute illness and injuries were 3.4 times more likely to incur CHE. In fact, households with the increased burden of acute illness and injury episodes were likely to face CHE by 2.3 times compared to those who did not have such burden. The treatment cost could easily exceed the CTP of households, especially of households with the poor economic condition, when they unexpectedly purchase healthcare by OOP. The household illness episode was considered in this study. This consideration holds explanation that the cost of each illness episode ultimately falls into the household healthcare expenditure as pointed by Sauerborn et al. [36].

The household economic condition was another key driver of CHE in Nepal. Households located in 1st and 2nd quartiles were almost equally vulnerable to CHE compared to the 4th quartile. Households from 4th quartile reported not only more episodes of illness but also greater CTP for their illness. However, even smaller healthcare expenditure was catastrophic to the poor households. This finding is consistent with earlier studies [28].

Households in the far-western region were more likely to incur CHE. This unique finding for Nepal holds two possible explanations. First, the far-western region has low development indicators in comparison to other regions [16]. In this study, more than three-quarters of households in the far-western region belonged to the poor quartile. This shows the economic status of the far-western region is not strong. This finding with some variation can be compared to the finding from the Chinese study [21]. Second, as this region is near to the Indian border, people might often commute to the Indian side for their treatment. This practice could potentially increase OOP healthcare expenditure of their households.

Households with educated household head were 27% less likely to incur CHE while households with children had no significant effect on CHE in Nepal. This study showed that households with literate head were less likely to incur CHE. Two reasons could explain this finding. First, household with literate household head might be aware of their health behaviour. Grossman theorizes that education brings health awareness [37]. Educated household heads are more cautious on their own health behaviour and that of their family members. In the long run, such households are likely to practice preventive behaviour to avoid illness which would further prevent catastrophic expenses in healthcare. This finding is consistent with a Tanzanian study [32]. Second, improved literacy could also lead to greater income generation. Improved income potentially leads to improved health as described in the published literature [38]. Nonetheless, households with children (≤ 5 years) had no significant association with CHE. Maternal-and-child health program is a priority program of the GoN. Services like immunization, treatment of childhood illness are provided free of cost [14]. Although the effects of maternal-and-child health programs were not analysed, we suppose, those free services might have contributed in reducing childhood related healthcare expenditure.

### Limitations of the study

The findings of this study should be considered in the context of a few major shortcomings. First, though limited, the presence of public subsidies cannot be denied in Nepal. However, the survey data used had no variables reflecting the utilization of those schemes. Second, strategies adopted by households to cope CHE was not analysed. A qualitative study is preferred to explore household coping strategies. This study warrants future studies to explore the strategies adopted by households to cope CHE. Third, although access to the closest health facility was seen, no further analysis of healthcare delivery side was done because of the limited variables in the NLSS data. Finally, the competing approach and threshold to measure CHE might yield different values of CHE. However, this study applied the standard method to measure CHE.

### Policy implications and recommendations

The commitment of the GoN to global community to achieve UHC by 2030 against the backdrop of limited fiscal space is a daunting challenge [13]. This study intends to bring CHE in the forefront of policy discussion as Nepal aims to introduce social health security scheme (SHSS) (technically called Social Health Insurance) to attain UHC [14]. In the long run, increasing the government’s share to finance healthcare is considered as obvious policy [35, 39]. Meanwhile, findings of this study can help locate the vulnerable households to lower CHE in Nepal. In light of its findings, this study recommends to: a) prioritize groups and regions strategically when introducing any risk-pooling mechanisms; and b) intensify interventions to improve literacy rate.

This study clearly showed that households in poorer quartiles and regions suffered from CHE. For instance, households in the far-west regions were from the poor quartile and were very likely to face CHE. So, this study recommends prioritizing the vulnerable households and regions. The far-western region could be selected as a pilot region for the SHSS. Also, unexpected expenditure for treating acute illness and injuries had more tendency to be catastrophic to the household in Nepal. Therefore, management of such morbidities should be the goals of policy-makers. Management could be done through improving general literacy rate in the country. This leads to our next recommendation: tailored interventions to improve literacy rate could be beneficial to manage acute illness and injuries in Nepal. An Indian study also reported that increasing literacy, especially among female member of a household, was advantageous in reducing catastrophic OOP health expenditure [40]. Interventions to improve literacy could be helpful in managing illnesses and lowering CHE in Nepal.

## Conclusions

The cumulative incidence rate of CHE was 10.3% per month in Nepal. CHE was concentrated in the poorer quartiles and far-western region. Furthermore, this study demonstrated that increased illness episodes in a household triggered CHE. CHE was also influenced by household’s regional location, economic status, chronic illness, acute illness, and education of household head. Findings of this study underscore the importance of incorporating efforts to effectively prioritize the vulnerable households and improve literacy with the current endeavours of the GoN. UHC is a health policy priority, globally. Policy recommendations offered by this study could be of use to lower the CHE and facilitate UHC in Nepal and countries with similar socio-economic context.

### Additional file

Additional file 1: Plots demonstrating the diagnostic check of the model 1. **Figure S1.** (A-F): Plots demonstrating the diagnostic check of the model 1. The additional file consists six graphs, A-F. A: Plot of Pearson residuals versus the predicted probability of catastrophic health expenditure. B: Plot of Pearson residuals versus household number. C: Plot of deviance residuals versus the predicted probability of catastrophic health expenditure. D: Plot of deviance residuals versus household number. E: Plot of leverage versus the predicted probability of catastrophic health expenditure. F: Plot of leverage versus household number. (DOCX 943 kb)

## Appendix

Equations used in the measurement of CHE

Main variables:

OOP = Out-of-pocket health expenditure

hh_exp = Household consumption expenditure

food_h = Food expenditure

eqsize = Equivalent household size

hhsize = Household size

eqfoodh = Equivalent food expenditure

pl = Poverty line

se = Subsistence spending

poorh = poor household

CTP = Capacity to pay

CHE = Catastrophic health expenditure

Steps:

Generate the equivalent household size for each household as

eqsize = hhsize^ β [The value of β (which is a household scale multiplier) used was 0.56 [4]. The value of 0.56 is obtained in from a regression equation based on 59 countries: ln(food_h) = ln(k) + β ln(hhsize) + ∑ γicountry]

Estimate equivalent food expenditure (eqfoodh) as

eqfoodh = foodh/eqsize

Identify the food expenditure shares of total household expenditure that are at the 45 th and 55 th percentile across the whole sample, name these two variables as foodh45 and foodh55.We used household weight (wh) in the percentile calculation. Calculate the average of food expenditure of the households in the 45th to 55th percentile range to obtain the subsistence expenditure per (equivalent) capita, which is also the poverty line (pl).

$$ \mathrm{pl}=\sum 45\mathrm{th}\ \mathrm{to}\ 55{\mathrm{th}}^{\ast}\left(\mathrm{eqfoodh}/\sum \mathrm{wh}\ \left(45\mathrm{th}\ \mathrm{to}\ 55\mathrm{th}\right)\right)\operatorname{}\mathrm{where}\ \mathrm{foodh}45<\mathrm{eqfoodh}<\mathrm{foodh}55 $$

Calculate Subsistence spending as, se = pl*eqsize

{A household is regarded as poor (poorh) when its total household expenditure is smaller than its subsistence spending. Poorh = 1 if hh_exp < se, otherwise Poorh = 0}

Next, calculate capacity to pay (CTP) as,

$$ \mathrm{CTP}=\mathrm{hh}\_\exp \hbox{-} \mathrm{se}\ \mathrm{if}\ \mathrm{se}<=\mathrm{foodh};\mathrm{CTP}=\mathrm{hh}\_\exp \hbox{-} \mathrm{foodh}\ \mathrm{if}\ \mathrm{se}>\mathrm{foodh} $$

Finally, calculate catastrophic health expenditure (CHE) as,

$$ \mathrm{CHE}=1\ \mathrm{if}\ \mathrm{OOP}/\mathrm{CTP}>=0.4,\mathrm{otherwise}\ \mathrm{CHE}=0 $$

## Abbreviations

CBS: Central Bureau of Statistics- Nepal
CHE: Catastrophic health expenditure
CI: Confidence interval
CTP: Capacity to pay
GoN: Government of Nepal
LR: Likelihood ratio
NLSS-III: Nepal living standards survey- third
NRs: Nepali Rupees
OOP: Out-of-pocket
OR: Odds ratio
p: *p*-value
PSU: Primary sampling unit
SDG: Sustainable development goal
SHSS: Social health security scheme
UHC: Universal health coverage
VIF: Variance inflation factor
χ^2^: Chi square

## Footnotes

1 Risk pooling mechanism in health system is a function which ensures that the risk related to financing health care interventions is shared by all the members of the pool. The major objective of risk pooling is to distribute the financial risk associated with health care interventions for which there is uncertain need [2, 3].
