# Supplementary material for: Cumulative incidence, distribution, and determinants of catastrophic health expenditure in Nepal: results from the living standards survey
Source: Int J Equity Health. 2018 Feb 14;17:23. doi: 10.1186/s12939-018-0736-x (PMC5813388; doi:10.1186/s12939-018-0736-x)
Supplement: Supplementary file 1 — Plots demonstrating the diagnostic check of the model 1. Figure S1. (A-F): Plots demonstrating the diagnostic check of the model 1. The additional file consists six graphs, A-F. A: Plot of Pearson residuals versus the predicted probability of catastrophic health expenditure. B: Plot of Pearson residuals versus household number. C: Plot of deviance residuals versus the predicted probability of catastrophic health expenditure. D: Plot of deviance residuals versus household number. E: Plot of leverage versus the predicted probability of catastrophic health expenditure. F: Plot of leverage versus household number. (DOCX 943 kb) [file 12939_2018_736_MOESM1_ESM.docx]

ADDITIONAL FILE 1


 A B


 C D

 E F

Figure S1(A-F): Plots demonstrating the diagnostic check of the model 1.

A: Plot of Pearson residuals versus the predicted probability of catastrophic health expenditure.

B: Plot of Pearson residuals versus household number.

C: Plot of deviance residuals versus the predicted probability of catastrophic health expenditure.

D: Plot of deviance residuals versus household number.

E: Plot of leverage versus the predicted probability of catastrophic health expenditure.

F: Plot of leverage versus household number.
